# Antiretroviral treatment coverage in a rural district in Tanzania – a modeling study using empirical data

**DOI:** 10.1186/s12889-015-1460-8

**Published:** 2015-02-27

**Authors:** Francis Levira, Abela Mpobela Agnarson, Honorati Masanja, Basia Zaba, Anna Mia Ekström, Anna Thorson

**Affiliations:** Data Analysis Cluster, Ifakara Health Institute, Plot 463, Kiko Avenue, Mikocheni, P O Box 78378, Dar es salaam, Tanzania; Department of Public Health Sciences/Global Health (IHCAR), Karolinska Institutet, Stockholm, Sweden; Department of Population Health, London School of Hygiene and Tropical Medicine, London, UK; Department of Infectious Diseases, Karolinska University Hospital, Stockholm, Sweden

**Keywords:** AIDS, ART, ARV, Coverage, HIV, Antiretroviral, Treatment, Tanzania, Rufiji

## Abstract

**Background:**

The Tanzanian Government started scaling up its antiretroviral treatment (ART) program from referral, regional and district hospitals to primary health care facilities in October 2004. In 2010, most ART clinics were decentralized to primary health facilities. ART coverage, i.e. people living with HIV (PLHIV) on combination treatment as a proportion of those in need of treatment, provides the basis for evaluating the efficiency of ART programs at national and district level. We aimed to evaluate adult ART and pre-ART care coverage by age and sex at CD4 < 200, < 350 and all PLHIV in the Rufiji district of Tanzania from 2006 to 2010.

**Methods:**

The numbers of people on ART and pre-ART care were obtained from routinely aggregated, patient-level, cohort data from care and treatment centers in the district. We used ALPHA model to predict the number in need of pre-ART care and ART by age and sex at CD4 < 200 and < 350.

**Results:**

Adult ART coverage among PLHIV increased from 2.9% in 2006 to 17.6% in 2010. In 2010, coverage was 20% for women and 14.8% for men. ART coverage was 30.2% and 38.7% in 2010 with reference to CD4 criteria of 350 and 200 respectively. In 2010, ART coverage was 0 and 3.4% among young people aged 15–19 and 20–24 respectively. ART coverage among females aged 35–39 and 40–44 was 30.6 and 35% respectively in 2010. Adult pre-ART care coverage for PLHIV of CD4 < 350 increased from 5% in 2006 to 37.7% in 2010. The age-sex coverage patterns for pre-ART care were similar to ART coverage for both CD4 of 200 and 350 over the study period.

**Conclusions:**

ART coverage in the Rufiji district is unevenly distributed and far from the universal coverage target of 80%, in particular among young men. The findings in 2010 are close to the most recent estimates of ART coverage in 2013. To strive for universal coverage, both the recruitment of new eligible individuals to pre-ART and ART and the successful retention of those already on ART in the program need to be prioritized.

## Background

In recognition of the positive impact of antiretroviral treatment (ART) on HIV-associated survival, the Tanzanian Government, in collaboration with international organizations and donor agencies, introduced a treatment program in October 2004 aimed at treating all eligible AIDS patient with antiretroviral drugs (ARV) [1-5]. ART scale-up in Tanzania started in 2004 with 96 care and treatment centers (CTC) which grew to about 1100 in 2010 [6]. The number of PLHIV enrolled on ART was 23,951 in December 2005, reached approximately 384,816 by 2010 [6,7]. AIDS-related mortality and new HIV infection decreased by 41% and 46% between 2005 and 2013 respectively [8]. Recent evidence shows in addition to treating AIDS patients, ART is potential for preventing further spread of HIV virus [9-11]. Slow progress in ART enrollment in many countries in resource-limited setting prompted a political declaration on HIV and AIDS in 2011 aimed at “Intensifying efforts to eliminate HIV and AIDS and reaffirming commitment towards the 2001 and 2006 goals of universal access to comprehensive prevention, treatment, care and support” [12,13]. Several global initiatives followed the UN declaration includes “Treatment 2015” aimed at reaching an estimated 15 million people with antiretroviral therapy by 2015 [14] and “UNAIDS 90-90-90” aimed to achieve the following by 2020; 90% of all PLHIV know their HIV status, 90% of all people with diagnosed HIV infection receive sustained antiretroviral therapy and 90% of all people receiving antiretroviral therapy have viral suppression [15].

ART treatment coverage is one of the key indicators used by program managers in assessing health system performance and community engagement in treating and preventing HIV. It is measured as PLHIV on combination treatment as a proportion of those in need of treatment [16]. The definition of the number of people in need of ART has varied over the years with changes in WHO guidelines (<200 CD4 in 2006, <350 in 2010 and <500 in 2013). Changes in the guidelines came as a result of emerging evidence from clinical trials of improved survival upon early ART initiation. In Tanzania, an eligibility criterion of CD4 count < 200 was adopted when the treatment program started in 2004 until 2012 when changes to adopt the 2010 World Health Organization (WHO) guideline of CD4 < 350 were made. An estimate from WHO showed adult coverage was 49% under CD4 < 200 in 2006. Under CD4 < 350 adult ART coverage was 32% in 2009, 40% in 2011 and 68% in 2012 [16].

Evaluation of the efficiency of ART programs through ART coverage has been hampered by a lack of reliable statistics on who is on treatment and who is eligible for treatment in most resource-limited countries. A substantial proportion of PLHIV are not aware of their HIV status and ART eligibility due to low HIV testing rates, therefore the ideal number of those in need of treatment cannot directly be obtained unless mathematical models are applied. The absence of a comprehensive information system to document exact number of PLHIV on treatment, ART adherence, drop-out and mortality makes the ART coverage estimation uncertain. UNAIDS developed the Spectrum model, a constantly updated, user-friendly software program which predicts the theoretical need for ART at national level by projecting distribution of expected CD4 counts and past HIV incidence in the population among other parameters [17,18]. However, some of the Spectrum’s model input parameters, such as the distribution of CD4 counts and HIV incidence, are rarely available at national and sub-national level and may lead to less reliable estimates of treatment coverage. This gap in valid program data, especially at sub-national level such as district, may compromise strategic priority and target settings in this era of power decentralization to the districts. So far, regional and district level data have not been well utilized in generating local level estimates of treatment coverage for district level planning. Recently, UNAIDS used all PLHIV as the denominator to calculate ART coverage. This is due to the fact that national ART initiation guidelines do not always match global guidelines, making country comparisons and time trend analysis of little value [19]. In 2013, UNAIDS estimated ART coverage for adults living with HIV regardless of CD4 count in Tanzania to be 41% [8]. A review of ART initiation criteria in the national guidelines for 94 countries (representing 86% of global HIV burden) showed that only seven countries from the developed world initiate ART irrespective of CD4 cell count [10]. Alternative methods to estimate the number of PLHIV in need of treatment for countries that have not adopted a “test and treat” policy are needed to evaluate whether programs have achieved their coverage target based on their recommended ART initiation policy.

The ALPHA network (The Analyzing Longitudinal Population-based HIV/AIDS data on Africa) has developed an alternative model which requires only age-specific mortality patterns of PLHIV to predict ART need for different CD4 initiation criteria [20].

The model estimates the proportion in need of ART, by sex and age group, among PLHIV who would be expected to die within a pre-specified period in the absence of ART. The pre-specified period is equivalent to CD4 count cut-offs; thus the model can be adopted for different countries or local policy guidelines of ART eligibility. The optimal period from ART initiation to death is an important parameter in deciding when to start ART in most policy guidelines. The model takes into account age-related disease progression and cumulative estimated treatment need due to the improved survival of those accessing treatment [21]. Model estimates can then be applied on national or sub-national populations of PLHIV to estimate the number in need of ART. Estimates of the number of HIV-positive individuals can be derived from representative HIV prevalence surveys available in many resource-limited countries. The additional advantage of the ALPHA model is its capability to estimate the proportion of HIV individuals in need of pre-ART care. Pre-ART care coverage is the ratio of PLHIV who are not eligible for ART (CD4 count > policy recommendation), over all PLHIV in the population. People on pre-ART care are enrolled for CD4 count monitoring, prophylaxis, nutritional advice and counseling. Early enrolment to pre-ART care is a gateway to appropriate timing of ART initiation, therefore pre-ART care coverage is an important indicator of the future success of national and regional ART program implementation [22,23].

We aimed to estimate adult pre-ART care and ART coverage by age and sex in the rural Rufiji district in Tanzania in line with the WHO guideline of 2006 and 2010 and with PLHIV using routinely aggregated patient-level cohort data from 2006–2010.

## Methods

### District population characteristics

Rufiji is one of six administrative districts in the Pwani Region of Tanzania approximately 180 km south of Dar es Salaam, the country’s largest city. The district has a population of approximately 217000 according to the most recent national census conducted in 2012 [24]. The regional literacy rate in the district is 73.6 and the majority of the population works in agriculture. Between 2008 and 2012, HIV prevalence among males in the region decreased from 4.2 to 2.1, increased among females from 8.4 to 9.2 and decreased in the general population from 6.7 to 5.9 according to the Tanzania HIV/AIDS and Malaria Indicator Survey (THMIS) of 2007/08 and 2012 [25,26]. There is considerable stigma directed at people living with HIV and those on ART in the district as described in detail in previous studies [27,28].

### Number of PLHIV in need of treatment

**Figure 1:**
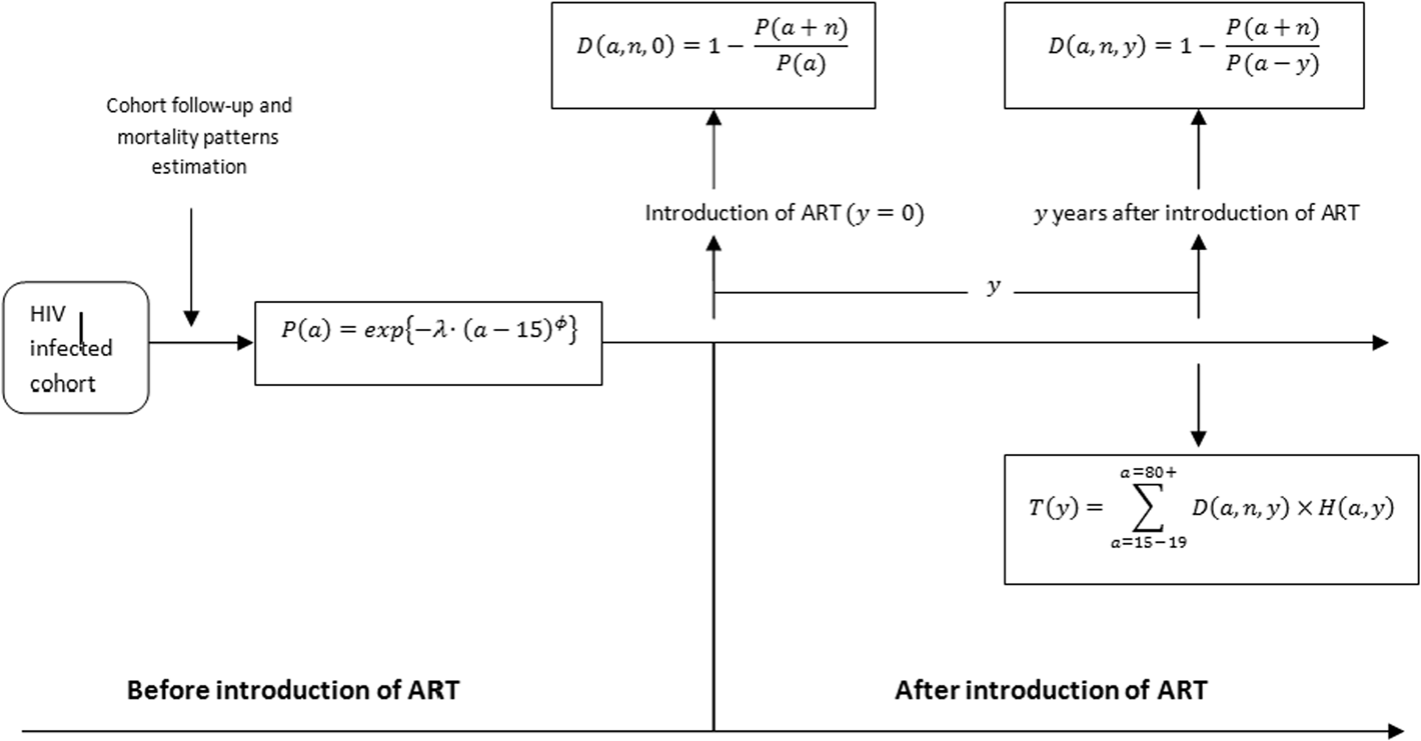
**Graphical summary of ALPHA model for estimating ART need.**

The ALPHA model estimation process is described in Figure 1. It begins with estimation of parameters describing age-specific mortality patterns of PLHIV in the absence of treatment from Weibul model. *P*(*a*) is survival probabilities for HIV-positive individuals from 15 years of age to age *a* years. Parameters describing age-specific mortality patterns are: overall mortality level (*λ*); and patterns of increase in mortality with age (*ϕ*). The model parameters can be obtained from studies conducted in settings where the ART estimation is being conducted or borrowed/adopted from studies that have been conducted in places with comparable population characteristics. *P*(*a*) is then applied to estimate *D*(*a*, *n*, 0)which is the probability of dying in the next *n* years for an HIV-infected person aged *a* in a population where ART is not yet available (i.e. available for 0 years). The value of *n* can be conceptualized as the mean time from the ideal start of treatment to expected AIDS death in the absence of treatment. This value is equivalent to the minimum level of CD4 count for AIDS patients to start ART. In this article, we chose values of n = 3 and n = 6 years which are equivalent to a CD4 count of 200 and 350 used as the threshold in the national ART programs. Further details on estimated values for different CD4 count levels can be found elsewhere in the literature [17,18,29,30].

We adopted parameter estimates *λ* and *ϕ* from HIV cohort study from the Kisesa district in Tanzania were the value of *λ* = 0.0094 and *ϕ* = 1.59 were estimated. The methodology on how these parameters were estimated has been fully described in a previous paper [20,31].

District ART programs that enroll individuals who are expected to die in the next *n* years in the absence of treatment, need to enroll additional people every calendar year as PLHIV cohorts age and more people enter into the high death risk period *y* years following the start of ART. The proportion of PLHIV currently aged *a* who will need treatment is defined by *D*(*a*, *n*, *y*). The final output of the model *T*(*y*) is the proportion of HIV-positive individuals in need of treatment in year *y* following the introduction of an ART program who are expected to die in *n* years in the absence of treatment. Age-specific proportions needing ART for individuals aged 15 to 49 years for CD4 initiating criteria of 200 and 350 or less produced by ALPHA model are shown in Tables 1 and 2 respectively.

**Table 1:** **Maximum age-specific proportions of HIV-positive people’s need of ART in 2005–2010 using CD4 criteria of < 200**

	**2005**	**2006**	**2007**	**2008**	**2009**	**2010**
**Age**	**0**	**1**	**2**	**3**	**4**	**5**
15 - 19	0.094	0.111	0.124	0.131	0.135	0.136
20 - 24	0.151	0.190	0.224	0.253	0.277	0.296
25 - 29	0.192	0.243	0.288	0.328	0.364	0.395
30 - 34	0.226	0.285	0.338	0.386	0.428	0.465
35 - 39	0.255	0.321	0.380	0.432	0.478	0.520
40 - 44	0.280	0.352	0.415	0.471	0.521	0.564
45 - 49	0.303	0.380	0.447	0.505	0.556	0.602

**Table 2:** **Maximum age-specific proportions of HIV-positive people’s need of ART in 2005–2010 using CD4 criteria of < 350**

	**2005**	**2006**	**2007**	**2008**	**2009**	**2010**
**Age**	**0**	**1**	**2**	**3**	**4**	**5**
15 - 19	0.212	0.227	0.238	0.244	0.247	0.248
20 - 24	0.301	0.333	0.361	0.384	0.404	0.420
25 - 29	0.364	0.404	0.440	0.471	0.499	0.524
30 - 34	0.414	0.459	0.499	0.535	0.567	0.595
35 - 39	0.456	0.505	0.547	0.585	0.619	0.649
40 - 44	0.492	0.543	0.587	0.627	0.662	0.692
45 - 49	0.523	0.576	0.621	0.661	0.697	0.727

To calculate the actual number needing treatment, the proportion of PLHIV who will need ART is applied to the numerical age distribution of HIV-positive individuals (*H*(*y*)) identified in the population in a particular year, allowing for the number of years *y* that have passed since the start of ART availability. The overall needs for ART are calculated by summing the number in need of treatment across age groups. The difference between the number of all PLHIV and the number of PLHIV in need of treatment with specific CD4 counts criteria gives the number in need of pre-ART care. Transitions from pre-ART care to ART for patients started treatment are described in the cascade transition figure.

### Number of people living with HIV in the population

The total annual number of people living with HIV in the population was obtained as a product of the annual district population and HIV prevalence. District age and sex population from 2006 to 2010 were estimated from the projection of 2002 national census data.

National average age- and sex-specific HIV prevalence estimates from the Tanzania HIV/AIDS indicator survey of 2007/2008 for adults aged 15–49 years were used [26]. Country estimates were considered because district estimates are not available, regional estimate do not include age- and sex-disaggregated data and district prevalence data are facility-based with a risk of over estimating prevalence. There were few changes in HIV prevalence between 2007 and 2012 in Tanzania (http://www.dhsprogram.com/). The HIV prevalence for individuals aged 15–49 in the 2007/2008 survey was 5.8 while the most recent survey, conducted in 2012, reported a prevalence of 5.3 [25].

### Data collection

#### Number of HIV-infected individuals on treatment

Patient data for this analysis were derived from the CTCs that provided ART services in the Rufiji district at the time of data collection. In 2005, an ART scale-up pilot was performed at Mchukwi Mission Hospital in Rufiji. By 2010, the ART program had been extended to Utete District Hospital (2006), Nyaminywili Health Centre (2009), Nyamisati Dispensary (2009), Ikwiriri Health Centre (2010) and Kibiti Health Centre (2010). In 2010, the Pwani region, where Rufiji is situated, had 28 CTC units, 23,212 patients were enrolled in care and 9,985 had commenced ART [32].

Patient data were retrieved from care and treatment registries. The care register keeps records of basic information on clients who have not yet started ART. Once a patient starts on ART, they are transferred to the ART treatment register. The patient data are kept across registries and longitudinally identified using the 7-digit HIV patient national identifier. The data extraction from the CTC database was done from September 2009 to January 2011. Stata software version 12.0 (Stata Corp, College Station, TX) was used to organize and generate descriptive statistics of patients registered for care and treatment.

#### Ethics statement

The national ethics committee of Tanzania (Medical Research Coordinating Committee) and the Tanzania Commission for Science and Technology (COSTECH) approved the study with approval reference number NIMR/HQ/R.8a/VolIX/609.

## Results

### PLHIV enrolled on district pre-ART care and ART programs

A total of 139 and 148 HIV-positive individuals were enrolled on pre-ART care and ART programs respectively in 2006 following a pilot study in 2005. By 2010, a total of 819 and 914 individuals were registered in pre-ART care and ART programs, representing a six-fold increase over a four-year period. There were more people on ART than in pre-ART care and more females than males in both pre-ART care and ART programs. Details of the distribution by year, age and sex are shown in Tables 3 and 4.

**Table 3:** **PLHIV enrolled on ART by age, sex and calendar year**

	**Both**					**Female**					**Male**				
**Age**	**2006**	**2007**	**2008**	**2009**	**2010**	**2006**	**2007**	**2008**	**2009**	**2010**	**2006**	**2007**	**2008**	**2009**	**2010**
15-19	2	2	3	4	3	2	2	3	4	3	0	0	0	0	0
20-24	5	16	26	33	40	4	11	22	29	36	1	4	4	4	4
25-29	17	58	77	105	123	16	52	68	91	103	1	6	9	14	19
30-34	35	92	153	191	237	25	69	117	148	184	10	23	36	43	53
35-39	49	102	142	201	244	33	69	96	137	170	16	34	46	64	75
40-44	29	69	105	135	193	20	40	60	78	126	10	29	45	57	67
45-49	13	30	46	58	75	7	16	23	29	39	5	14	23	29	36
**Total**	**148**	**370**	**552**	**725**	**914**	**106**	**259**	**390**	**514**	**661**	**42**	**110**	**163**	**211**	**253**

**Table 4:** **PLHIV enrolled on pre-ART care by age, sex and calendar year**

	**Both**					**Female**					**Male**				
**Age**	**2006**	**2007**	**2008**	**2009**	**2010**	**2006**	**2007**	**2008**	**2009**	**2010**	**2006**	**2007**	**2008**	**2009**	**2010**
15-19	3	4	9	8	6	2	4	9	8	6	1	0	0	0	1
20-24	8	26	34	55	71	5	21	29	49	64	2	5	4	6	7
25-29	24	45	72	126	155	20	42	64	103	130	4	3	9	23	25
30-34	43	57	128	172	213	35	45	99	133	159	7	12	30	39	54
35-39	33	65	97	155	200	22	43	66	98	137	11	22	31	56	63
40-44	21	45	72	91	121	14	26	46	53	76	7	19	26	39	45
45-49	8	13	24	39	52	5	9	14	22	30	2	5	10	17	22
**Total**	**139**	**251**	**427**	**638**	**819**	**105**	**190**	**327**	**466**	**602**	**34**	**66**	**110**	**180**	**218**

### Transition from pre-ART to ART

We observe in cumulative a total of 1059 (38%) individual’s transition from pre-ART to ART cascade among 2758 individuals started pre-ART in the study area. The transition took on average 3 months and longer (3.7 months) for those initiated pre-ART care at an earlier stage of the disease (WHO stage 1) compared to those started at late stage (WHO stage 4) (2.3 months).

### PLHIV needing pre-ART care and ART

**Figure 2:**
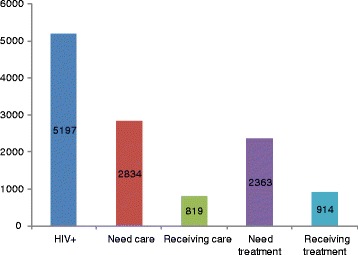
**Numbers of PLHIV in the cascade of care and treatment in 2010.**

The number of adults living with HIV needing ART irrespective of CD4 counts, CD4 of 350 and 200 between 2006 and 2010 are presented in Tables 5, 6 and 7 respectively. Compared to providing ART to all 5197 PLHIV in the district in 2010, the district could aim to provide ART to 3027 (42% less) if 2010 guidelines were in place and 2363 (54% less) with CD4 criteria of 200. The district provided pre-ART care to 819 and ART to 914 PLHIV in 2010 (Figure 2).

**Table 5:** **Number of HIV-positive people’s need of ART in 2005–2010 using of all HIV+ individual**

	**ALL**				
	2006	2007	2008	2009	2010
15 - 19	185	186	187	189	190
20 - 24	646	651	655	660	665
25 - 29	915	921	928	934	941
30 - 34	1162	1171	1179	1187	1195
35 - 39	1059	1066	1074	1081	1089
40 - 44	657	662	666	671	676
45 - 49	429	432	435	439	442
**Total**	**5053**	**5089**	**5124**	**5161**	**5197**
	**FEMALE**				
15 - 19	119	120	121	122	123
20 - 24	550	553	557	561	565
25 - 29	624	628	633	637	641
30 - 34	747	753	758	763	769
35 - 39	539	543	547	551	555
40 - 44	350	352	355	357	360
45 - 49	231	233	234	236	237
**Total**	**3160**	**3182**	**3205**	**3227**	**3250**
	**MALE**				
15 - 19	67	67	66	66	65
20 - 24	113	112	111	110	110
25 - 29	327	325	323	321	318
30 - 34	418	415	412	410	407
35 - 39	454	451	448	445	442
40 - 44	228	226	224	223	221
45 - 49	154	153	152	151	150
**Total**	**1762**	**1749**	**1737**	**1725**	**1713**

**Table 6:** **Number of HIV-positive people’s need of ART in 2005–2010 using CD4 criteria of < 350**

	**ALL**				
	**2006**	**2007**	**2008**	**2009**	**2010**
15 - 19	42	44	46	47	47
20 - 24	215	235	252	267	279
25 - 29	370	405	437	466	493
30 - 34	534	585	631	673	712
35 - 39	534	584	629	670	707
40 - 44	357	389	418	444	468
45 - 49	247	269	288	305	321
**Total**	**2299**	**2509**	**2700**	**2872**	**3027**
	**FEMALE**				
15 - 19	27	29	29	30	30
20 - 24	183	200	214	227	237
25 - 29	252	276	298	318	336
30 - 34	343	376	406	433	458
35 - 39	272	297	320	341	360
40 - 44	190	207	222	236	249
45 - 49	133	145	155	164	173
**Total**	**1400**	**1529**	**1645**	**1750**	**1844**
	**MALE**				
15 - 19	15	16	16	16	17
20 - 24	36	39	42	44	46
25 - 29	116	127	138	147	155
30 - 34	190	208	224	239	253
35 - 39	262	287	309	329	347
40 - 44	165	179	193	205	216
45 - 49	116	126	136	144	151
**Total**	**900**	**982**	**1057**	**1124**	**1186**

**Table 7:** **Number of HIV-positive people’s need of ART in 2005–2010 using CD4 criteria of < 200**

	**ALL**				
	**2006**	**2007**	**2008**	**2009**	**2010**
15 - 19	21	23	25	25	26
20 - 24	123	146	166	183	197
25 - 29	222	265	305	340	372
30 - 34	332	396	454	508	556
35 - 39	340	405	464	517	566
40 - 44	231	275	314	349	381
45 - 49	163	193	220	244	266
**Total**	**1431**	**1703**	**1947**	**2166**	**2363**
	**FEMALE**				
15 - 19	13	15	16	16	17
20 - 24	104	124	141	155	167
25 - 29	152	181	208	232	254
30 - 34	213	255	292	326	358
35 - 39	173	206	236	264	288
40 - 44	123	146	167	186	203
45 - 49	88	104	118	131	143
**Total**	**866**	**1031**	**1178**	**1311**	**1429**
	**MALE**				
15 - 19	7	16	9	9	9
20 - 24	20	58	27	30	33
25 - 29	70	198	96	107	117
30 - 34	118	322	162	181	198
35 - 39	167	429	228	254	278
40 - 44	107	256	145	161	176
45 - 49	77	184	104	115	125
**Total**	**566**	**1463**	**770**	**857**	**936**

### Annual ART coverage by sex

The overall ART coverage among PLHIV increased from 2.9% in 2006 to 17.6% in 2010 (Table 8). In 2010, the ART coverage among PLHIV was 20% for women and 14.8% for men. The adult ART coverage was 21.4% and 35.9% in 2010 with reference to CD4 criteria of 350 for males and females respectively. With reference to CD4 criteria of 200, adult ART coverage was 27.1% and 46.2% for males and females respectively. Coverage was consistently higher among women than among men over the study period.

**Table 8:** **ART coverage by sex, CD4 threshold and year**

	**Both**			**Male**			**Female**		
	**< 200**	**<350**	**HIV+**	**<200**	**<350**	**HIV+**	**<200**	**<350**	**HIV+**
2006	10.4	6.5	2.9	7.5	4.7	2.4	12.2	7.6	3.4
2007	21.7	14.7	7.3	7.6	11.2	6.3	25.2	17.0	8.2
2008	28.4	20.5	10.8	21.1	15.4	9.4	33.1	23.7	12.2
2009	33.5	25.2	14.0	24.6	18.7	12.2	39.2	29.4	15.9
2010	38.7	30.2	17.6	27.1	21.4	14.8	46.2	35.9	20.3

### Annual sex-specific pre-ART care coverage

The adult pre-ART care coverage for PLHIV of CD4 < 350 increased from 5% in 2006 to 37.7% in 2010. Similar to ART coverage, the pre-ART care coverage was almost twice as high among females compared to males over the study period (Table 9). The pre-ART care coverage for PLHIV with CD4 < 350 was higher than for PLHIV with CD4 < 200 since the majority of those considered in need of pre-ART care were shifted to those who needed ART. In the “test and treat” policy scenario, all PLHIV would be eligible for ART, which is why the pre-ART care coverage is zero.

**Table 9:** **Pre-ART care coverage by sex, CD4 threshold and year**

	**Both**			**Male**			**Female**		
	**<200**	**<350**	**HIV+**	**< 200**	**< 350**	**HIV+**	**< 200**	**< 350**	**HIV+**
2006	3.8	5.0	0	2.6	3.6	0	4.6	6.0	0
2007	7.4	9.7	0	2.4	7.6	0	8.8	11.5	0
2008	13.5	17.6	0	9.5	13.7	0	16.1	20.9	0
2009	21.3	27.9	0	16.6	24.2	0	24.3	31.5	0
2010	28.9	37.7	0	21.4	21.3	0	33.0	43.6	0

### Annual age- and sex-specific pre-ART care and ART coverage

**Figure 3:**
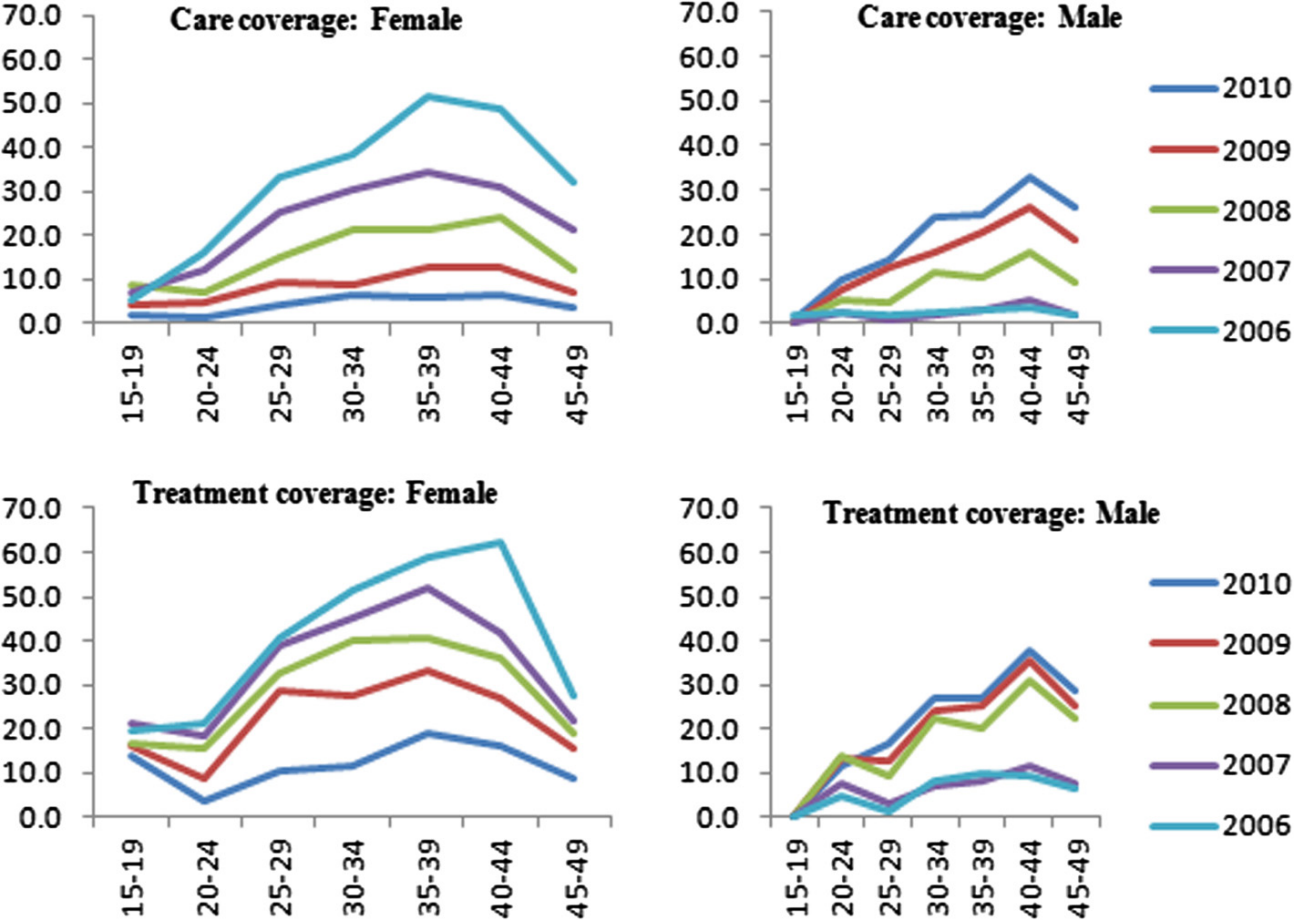
**Percent in need of care and treatment by age group and calendar year for CD4 < 200 criteria.**

ART coverage increased across all age groups and sexes between 2006 and 2010 (Figure 3 for CD4 < 200 in 2010). ART coverage among PLHIV was very low among young people aged 15 to 24 and in particular among men (Table 10). In 2010, treatment coverage among PLHIV was 1.7 and 5.9% for individuals aged 15–19 and 20–24 respectively. High coverage was observed among individuals aged 40–44 where females and males had coverage estimate of 35% and 30.3% respectively. ART coverage was zero among 15–19 year-olds and only 3.4% and 6% in young men in the groups 20–24 and 25–30 respectively in 2010.

**Table 10:** **ART coverage by sex, age and CD4 threshold**

	**CD4 < 200**														
	**Both**					**Female**					**Male**				
Age	2006	2007	2008	2009	2010	2006	2007	2008	2009	2010	2006	2007	2008	2009	2010
15-19	8.9	10.2	10.8	13.9	12.7	13.8	15.8	16.7	21.5	19.7	0.0	0.0	0.0	0.0	0.0
20-24	3.9	10.7	15.6	17.9	20.1	3.7	9.0	15.7	18.5	21.4	4.9	7.8	13.7	13.0	11.3
25-29	7.5	21.9	25.4	30.8	33.0	10.4	28.6	32.9	39.1	40.8	1.3	3.2	9.5	13.0	16.4
30-34	10.4	23.3	33.7	37.6	42.6	11.6	27.2	40.2	45.4	51.3	8.3	7.1	22.3	23.8	26.9
35-39	14.3	25.3	30.6	38.8	43.2	18.9	33.3	40.8	51.9	58.9	9.6	7.9	20.1	25.2	26.8
40-44	12.7	25.2	33.4	38.6	50.6	16.0	27.2	36.1	41.8	62.0	9.0	11.5	30.9	35.4	38.1
45-49	7.7	15.6	20.9	23.6	28.1	8.5	15.7	19.2	22.0	27.3	6.7	7.5	22.5	25.0	28.5
	CD4 < 350														
	2006	2007	2008	2009	2010	2006	2007	2008	2009	2010	2006	2007	2008	2009	2010
15-19	4.4	5.3	5.8	7.6	7.0	6.8	8.2	8.9	11.7	10.8	0.0	0.0	0.0	0.0	0.0
20-24	2.2	6.6	10.3	12.2	14.2	2.1	5.6	10.3	12.7	15.1	2.8	11.5	9.0	8.9	8.0
25-29	4.5	14.3	17.7	22.4	24.9	6.3	18.8	22.9	28.5	30.8	0.8	4.9	6.6	9.5	12.4
30-34	6.5	15.8	24.3	28.4	33.3	7.2	18.4	28.9	34.2	40.1	5.1	11.0	16.0	18.0	21.0
35-39	9.1	17.5	22.6	30.0	34.6	12.0	23.1	30.1	40.1	47.2	6.1	11.7	14.8	19.5	21.4
40-44	8.2	17.8	25.1	30.3	41.2	10.4	19.2	27.1	32.9	50.5	5.8	16.4	23.2	27.8	31.0
45-49	5.1	11.2	16.0	18.8	23.3	5.6	11.3	14.7	17.6	22.6	4.4	10.9	17.2	20.0	23.6
	All HIV +														
	2006	2007	2008	2009	2010	2006	2007	2008	2009	2010	2006	2007	2008	2009	2010
15-19	1.0	1.3	1.4	1.9	1.7	1.5	2.0	2.2	2.9	2.7	0.0	0.0	0.0	0.0	0.0
20-24	0.7	2.4	3.9	4.9	5.9	0.7	2.0	4.0	5.1	6.3	0.9	4.0	3.4	3.6	3.4
25-29	1.8	6.3	8.3	11.2	13.0	2.5	8.2	10.8	14.2	16.1	0.3	1.9	2.8	4.3	6.0
30-34	3.0	7.9	13.0	16.1	19.8	3.3	9.2	15.5	19.4	23.9	2.3	5.5	8.7	10.5	13.1
35-39	4.6	9.6	13.2	18.6	22.5	6.1	12.6	17.6	24.8	30.6	3.5	7.5	10.2	14.4	16.9
40-44	4.5	10.4	15.8	20.1	28.5	5.6	11.3	17.0	21.8	35.0	4.2	13.0	19.9	25.6	30.3
45-49	2.9	7.0	10.6	13.1	16.9	3.2	7.0	9.7	12.2	16.4	3.3	9.0	15.3	19.0	23.8

The distributions of pre-ART care coverage were similar to the pattern of ART coverage for all age groups and both sexes. As opposed to most high-income settings, pre-ART care coverage estimates were slightly lower than the treatment coverage values for all age groups and both sexes. More details on the distribution of pre-ART care coverage by sex, age and calendar year are given in Table 11.

**Table 11:** **Pre-ART care coverage by sex, age and CD4 threshold**

	**CD4 < 200x**														
	**Both**					**Female**					**Male**				
Age	2006	2007	2008	2009	2010	2006	2007	2008	2009	2010	2006	2007	2008	2009	2010
15-19	1.8	2.7	5.7	4.8	3.8	1.8	4.0	8.6	7.2	5.4	1.7	0.2	0.3	0.3	0.9
20-24	1.5	5.1	6.9	11.5	15.2	1.2	4.9	7.1	12.1	16.1	2.6	2.3	5.4	7.3	9.6
25-29	3.5	6.8	11.6	21.2	27.3	4.3	9.3	15.0	25.4	33.5	1.8	0.7	4.5	12.4	14.1
30-34	5.2	7.3	17.7	25.3	33.3	6.6	9.0	21.2	30.5	38.7	2.5	1.9	11.5	16.1	23.8
35-39	4.6	9.9	15.9	27.4	38.2	6.0	12.8	21.2	34.2	51.3	3.0	3.1	10.4	20.3	24.6
40-44	4.9	11.7	20.4	28.4	41.1	6.3	12.7	24.4	30.8	48.5	3.3	5.3	16.1	26.0	33.2
45-49	2.8	5.6	11.0	20.1	29.5	3.7	6.9	12.3	21.0	32.0	1.8	2.0	9.5	18.6	26.2
	CD4 < 350														
	2006	2007	2008	2009	2010	2006	2007	2008	2009	2010	2006	2007	2008	2009	2010
15-19	2.1	3.1	6.5	5.5	4.4	2.1	4.6	9.8	8.3	6.2	1.9	0.4	0.4	0.4	1.1
20-24	1.8	6.2	8.4	14.0	18.5	1.5	6.0	8.6	14.7	19.5	3.0	6.3	6.4	8.8	11.7
25-29	4.5	8.7	14.8	26.9	34.7	5.5	11.8	19.0	32.2	42.6	2.0	1.8	5.2	14.5	16.7
30-34	6.8	9.7	23.4	33.5	44.1	8.8	11.9	28.0	40.4	51.1	3.3	5.8	15.4	21.9	32.9
35-39	6.2	13.5	21.8	37.6	52.4	8.3	17.6	29.1	46.9	70.3	4.8	10.8	16.8	33.2	40.9
40-44	6.9	16.6	28.9	40.2	58.3	8.9	18.0	34.6	43.7	68.8	6.3	20.4	31.3	51.2	66.3
45-49	4.2	8.2	16.1	29.3	43.1	5.5	10.0	17.9	30.7	46.7	3.4	8.0	18.6	37.1	53.0

### ART coverage forecast

**Figure 4:**
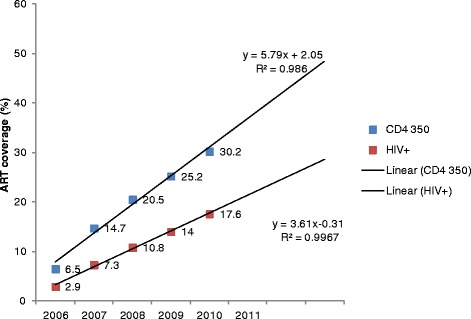
**Annual estimate and forecast of ART coverage at CD4 350 and PLHIV.**

Annual ART coverage was observed to increase in linear trends over the analysis period. Linear forecast of ART coverage at CD4 count < 350 was estimated at 42, 48 and 54% in 2011, 2012 and 2013 respectively (Figure 4). ART coverage among PLHIV was forecast to reach 25, 28 and 32% in 2011, 2012 and 2013 respectively.

## Discussion

Age- and sex-specific ART coverage are essential in evaluating progress and program implementation in the district. ART is not only relevant in treating AIDS patient but implemented as prevention strategy (TasP). We utilized district care and treatment data to estimate pre-ART care and ART coverage in a rural district of Tanzania. We provided age- and sex-disaggregated estimates of ART coverage to give district program managers a broader understanding of the coverage gap so that they could make informed decisions on resource allocation. National estimates of ART coverage are routinely reported separately for children (<15 years) and adults (15 years and above) which may be slightly less comparable to the coverage reported in this analysis (15–49 years). The district and national comparisons in this research may provide insights into trends and coverage levels and must be interpreted with caution. Our annual analysis of ART coverage in the rural district of Rufiji in Tanzania between 2006 and 2010 showed slow progress in scaling up and in reaching PLHIV with ART. Overall ART coverage among adult PLHIV increased from 3 to 18% while coverage based on CD4 criteria of < 350 increased from 6 to 30% between 2006 and 2010. Using ART eligibility that was operational during the study period (CD4 < 200), coverage increased from 10 to 38% between 2006 to 2010. The national estimate reported by WHO in 2009 was 32%, slightly higher than the estimated 25% in the district in the same year. Using the 2010 WHO guideline, the ART coverage forecast in the district reached 48 and 54% where the national estimate was 40 and 68% in 2011 and 2012 respectively. In 2013, WHO estimated ART coverage at 41% for adults PLHIV in Tanzania while projected district ART coverage for PLHIV was 32%, substantially lower than the national estimate. The findings from the analysis provide evidence of low ART coverage in the district, under the operational target of reaching PLHIV with a CD4 count < 200 and universal coverage.

Disaggregated analysis of ART coverage provides evidence that treatment-seeking behavior is driven by aging and sex. Results suggest that more than twice as many women compared to men were enrolled in ART. Women are generally diagnosed earlier with HIV because of HIV testing services offered during antenatal care visits [33]. ART coverage was consistently lower for males and young people over the study period. Remarkably low coverage among young people aged 15 to 24 and men in particular were recorded in this study. While the overall ART coverage was 18% in 2010, coverage among females aged 35–44 was above 30% and below 4% among young males under the age of 25. This is a cause for concern, given that men and women aged 15–24 years accounted for 22% of all new adult HIV infections in 2011, and this is the group least likely to know their HIV status and most likely to be sexually active, have multiple partners and reproduce [34,35]. An HIV testing study conducted in 2011 for sub-Saharan Africa showed that only 15% and 10% of young women and men respectively aged 15–24 years had been tested and had their HIV status confirmed [36]. In 2012, the HIV prevalence was estimated at 3.4% among women and 1.4% among men aged 15–24 years in Tanzania [32,37].

Early enrolment to pre-ART care is a gateway to appropriate timing of ART initiation and pre-ART care coverage is therefore an important indicator of the future success of national and regional ART program implementation. The fact that the majority who start on ART in Tanzania do so at advanced stage of AIDS is likely to be the primary contributor to high attrition and mortality rates in the first year of the ART program [38]. Loss to follow-up (up to 36%) and high mortality (up to 15%) have been observed in the past three years following ART initiation in Tanzania [38].

Slow progress was made in the proportion of HIV-positive individuals in pre-ART care. Coverage was observed to increase from 5% in 2006 to 38% in 2010 under CD4 eligibility of < 350. The distribution in terms of age and sex was similar to ART coverage characterized by low coverage for young people and men in particular.

Scaling up ART coverage to 80% of PLHIV by 2015 entails increasing the number of people initiating ART every year while keeping those already started on treatment adherent to ART. Further, decentralizing service delivery to primary health care facilities and reaching the young population with counseling and testing services are key to expanding coverage. The low ART coverage in this rural Tanzanian district demonstrates the challenge in meeting global targets and calls for an adjustment of district pre-ART care and ART programs in order to fit local realities and priorities. The current financial context and severe lack of human resources are particularly challenging [37,39].

Coverage estimates depend considerably on an accurate estimation of the number of PLHIV in surveys and a comprehensive information system to keep track of those enrolled on ART. HIV prevalence surveys in Tanzania are often confined to individuals aged 15 to 49 years and therefore the numbers of PLHIV among children younger than 15 and adults older than 50 years are not known for certain. As a result, this study excludes coverage estimation for children and elderly. Due to a lack of reliable data on HIV prevalence in the district, we used national estimates of age- and sex-specific HIV prevalence to calculated the number of PLHIV, and thus run the risk of over-estimation if the national average is far from the true prevalence in the study area. Our basic model assumptions could bias the results. If a different survival assumption had been applied (or eligibility for treatment set at a higher CD4 count, which is currently indeed the case in global guidelines), the proportion in need of treatment would be higher than the current estimates. The second model assumption is that there is no sex difference in mortality. This is less likely to be violated based on existing empirical data, but a higher overall mortality level for one sex will lead to a higher treatment need across all age groups. To estimate the cumulative number in need of treatment year by year, the model assumes that treatment needs for prior years have been fully satisfied, thus estimating maximum HIV care and ART coverage. Based on this assumption, the real coverage may be slightly lower if substantial proportion of PLHIV is not enrolled every year. There is a reduced version of the model which estimates reduced needs (minimum need) taking into consideration program success in enrolling all HIV-positive individuals [21]. This study addresses priority areas that could assist policymakers and implementers to expand access to pre-ART care and ART beyond currently achieved targets in particular for young people aged 15–24 years and for men. This study has shown that behind low treatment coverage is very low pre-ART care coverage, a factor which is an important gateway to ART treatment and a better treatment outcome. A balanced approach should address both the recruitment of new eligible individuals to pre-ART care and ART as well as the successful retention of those already on ART in the program.

## Conclusion

ART coverage in the Rufiji district in rural Tanzania is unevenly distributed and far from the universal coverage target of 80%, in particular among young men. Connected to the low ART coverage is the low pre-ART care coverage observed in this study. Taking the most recent WHO estimate of 2013 into consideration, there has been little progress in keeping up with ART needs since 2010. Low pre-ART care coverage may be highly associated with low testing rates and stigma towards individuals with AIDS as observed elsewhere in Tanzania. Although attrition analysis was not the aim of this study, we observed a substantial proportion of drop-out in both pre-ART care and ART programs which also may have contributed to the observed low coverage. To strive for universal coverage, both the recruitment of new eligible individuals to pre-ART and ART as well as the successful retention of those already on ART in the program need to be prioritized.

## Abbreviations

AIDS: Acquired immune deficiency syndrome
AIS: HIV/AIDS indicator survey
ALPHA: The analyzing longitudinal population-based HIV/AIDS data on Africa
ANC: Antenatal care
ART: Antiretroviral treatment
ARV: Antiretroviral drugs
CTCs: Care and Treatment Centers
HIV: Human Immunodeficiency Virus
HR: Hazard ratio
LTFU: Lost to follow up
PLHIV: People living with HIV
RHDSS: Rufiji health and demographic surveillance system
SSA: Sub-Saharan Africa
UNAIDS: The joint united nations programme on HIV and AIDS
WHO: World Health Organization
